# Balancing innovation and affordability in relapsing-remitting multiple sclerosis: a budget impact analysis from Saudi Arabia

**DOI:** 10.3389/fpubh.2025.1713092

**Published:** 2025-12-01

**Authors:** Ahmed Al-Jedai, Hajer Almudaiheem, Faisal Al-Suweidan, Mohamed al Jumah, Ahmed al-Thobaiti, Fahad Alzureiqan, Wejdan Abu Ras, Fahad M. Al-Dosari, Yaser Al Malik, Pratik Dhopte, Rita Ojeil

**Affiliations:** 1 Colleges of Medicine and Pharmacy, Alfaisal University, Riyadh, Saudi Arabia; 2 The Saudi Society of Clinical Pharmacy, Riyadh, Saudi Arabia; 3 Division of Neurology, Department of Medicine, Security Forces Hospital, Riyadh, Saudi Arabia; 4 King Abdullah International Medical Research Center (KAIMRC), Riyadh, Saudi Arabia; 5 Department of Neurology, King Saud bin Abdulaziz University for Health Sciences, Riyadh, Saudi Arabia; 6 King Saud Medical City, Riyadh, Saudi Arabia; 7 Drug Policy & Regulation Unit, Ministry of Health, Riyadh, Saudi Arabia; 8 Therapeutic Affairs Deputyship, Saudi Ministry of Health, Riyadh, Saudi Arabia; 9 Department of Clinical Pharmacy, King Saud Medical City, Riyadh First Health Cluster (R1), Ministry of Health, Riyadh, Saudi Arabia; 10 College of Medicine, King Saud bin Abdulaziz University for Health Sciences, Riyadh, Saudi Arabia; 11 Department of Neurology, King Abdulaziz Medical City, National Guard Health Affairs, Riyadh, Saudi Arabia; 12 Carexso, Dubai, United Arab Emirates; 13 PDC-CRO, Dubai, United Arab Emirates

**Keywords:** budget impact analysis, relapsing-remitting multiple sclerosis, managed entry agreements, disease-modifying therapies, multiple sclerosis

## Abstract

**Introduction:**

Relapsing-Remitting Multiple Sclerosis (RRMS) is the most prevalent form of multiple sclerosis, characterized by episodic neurological deterioration and recovery. The rising burden of RRMS in Saudi Arabia, underscores the need for cost-effective treatment strategies. This study evaluates the economic impact of disease-modifying therapies (DMTs) for RRMS from a national healthcare payer perspective, with a focus on the role of managed entry agreements (MEAs) in optimizing affordability.

**Methods:**

A budget impact analysis was conducted using an Excel-based model over a 5-year period. The model incorporated real-world uptake scenarios derived from expert opinion and hypothetical 100% uptake. The eligible population was stratified by treatment status and disease severity. Costs included drug acquisition, administration, monitoring, relapse management, and adverse event management. Scenarios with and without MEAs were analyzed to estimate net budget impact.

**Results:**

MEAs were associated with reduced treatment and relapse-related costs across all patient subgroups. Ofatumumab showed favorable economic profiles under MEA conditions, while cladribine (Mavenclad®) emerged as the most cost-efficient option. Siponimod (without MEA) in Active RRMS naïve (100% uptake) resulted in the highest overall expenditure, totaling SAR 732,484,106.83, highlighting the importance of strategic pricing and reimbursement models for high-cost DMTs in RRMS.

**Conclusion:**

This is the first comprehensive exploratory economic evaluation comparing first-in-class RRMS therapies in Saudi Arabia. Findings support the adoption of MEAs and performance-based reimbursement to ensure sustainable RRMS care in resource-constrained settings.

## 1 Introduction

Multiple Sclerosis (MS) is an immune-mediated, chronic, progressive neurodegenerative disorder of the central nervous system (CNS). It is characterized by axonal lesions, demyelination and inflammation (1, 2). Before definite MS develops, patients may show clinically isolated syndrome, a first MS-like neurological event, or radiologically isolated syndrome, where magnetic resonance imaging (MRI) reveals MS-type lesions without symptoms. These stages can be further described by disease activity (relapses or new MRI lesions) and progression, providing a complete description of the MS spectrum (3).

MS is broadly classified into two major clinical forms based on disease activity and progression. These include relapsing MS and progressive MS in either active or inactive form. The most common type, affecting around 80% of patients is relapsing-remitting MS (RRMS), which is defined by periods of symptoms flare-ups followed by recovery, and may later evolve into secondary progressive MS (SPMS), where disability gradually worsens between relapses. About 15% of cases are primary progressive MS (PPMS), in which symptoms progress steadily from the start (4, 5).

Globally, MS incidence is estimated at 2.5 cases per 100,000 individuals, with higher rates observed in countries such as Canada, Australia and parts of Europe (3.0–13.4 per 1,000,000) (6). In Saudi Arabia (SA), according to the 2020 update, the prevalence of MS in the Eastern Mediterranean Region (EMR) has shown a significant (37.5%) increase in prevalence from 24 per 100,000 individuals in 2013 to 33 per 100,000 in 2020. This escalating prevalence underscores a growing public health burden and the urgent need for strategic healthcare planning across the region. In 2020, SA reported an MS prevalence of 41 per 100,000 (7), surpassing the regional average but remaining lower than other Gulf countries. For context, the global MS prevalence stands at 37 per 100,000 individuals, positioning the EMR close to global figures while also reflecting notable intra-regional variability (4, 8). The disease affects women more commonly than men in SA, consistent with regional and global patterns (9).

Treatment in MS focuses on controlling inflammation to reduce relapses and radiological activity and to slow disability progression (10). Disease-modifying therapies (DMTs) are central to RRMS management and include multiple classes with distinct mechanisms and route of administration (1). Importantly, new CNS lesions may form at rates 10–20 times higher than clinical relapses (11). RRMS can be stratified based on disease activity (active, highly active and aggressive) and treatment history (naïve or treatment). *Active, Highly Active*, or *Aggressive RRMS* are defined by clinical relapses or MRI activity within 12 months (12). *Active RRMS* is characterized by relapses and/or MRI evidence of disease activity. *Highly Active RRMS* involves relapses, MRI progression, or worsening disability despite ≥1 year of therapy, often requiring treatment escalation or switch. *Aggressive RRMS* is defined by one or more of the following: Expanded Disability Status Scale (EDSS) score ≥4 within 5 years of onset, two or more relapses with incomplete recovery in the past year, multiple new or enlarging T2 or gadolinium-enhancing MRI lesions, or lack of clinical response to at least one DMT over a year (4). Recently, Middle East North Africa Committee for Treatment and Research in Multiple Sclerosis (MENACTRIMS) consensus, 2024, stratified RRMS by disease activity as moderately, highly and rapidly evolving aggressive RRMS: moderately active RRMS are cases with activity greater than mild but not meeting higher thresholds; highly active RRMS is indicated by features such as ≥2 relapses in the previous year, severe or incompletely recovered relapses, a high T2 lesion load (≥10 lesions, especially spinal or infratentorial), or multiple gadolinium-enhancing lesions; and the severe subgroup, termed rapidly evolving aggressive disease, is characterized by ≥2 disabling relapses with incomplete recovery in the past year with a high T2 lesion load (13) (Table 1).

**Table 1:** Clinical classification and key features of multiple sclerosis phenotypes.

MS subtypes	Definition/Features	Clinical course	Reference
Active RRMS	Clinical relapses and/or MRI activity (new/enlarging T2 lesions or contrast-enhancing lesions)	Relapsing	(3, 53, 54)
Naïve RRMS	Newly diagnosed RRMS patients who have not yet received DMT; typically, with recent clinical or radiologic activity	Relapsing	(53, 54)
SPMS	Transition from RRMS to gradual progression of disability, with or without superimposed activity	Progressive after relapsing phase	(3, 7, 55)
Active SPMS	SPMS with current disease activity (clinical relapses and/or MRI activity)	Progressive with activity	(3, 53, 56)
Non-active SPMS	SPMS without relapses or MRI activity	Progressive without activity	(3, 7)

MS, multiple sclerosis; RRMS, relapsing-remitting multiple sclerosis; SPMS, secondary progressive multiple sclerosis; DMT, disease-modifying therapies; MRI, magnetic resonance imaging.

Given the clinical and epidemiological burden of RRMS, evaluating the economic impact of DMTs is essential for informed decision-making and sustainable resource allocation in SA. Comparing with and without managed entry agreements (MEAs) help in assessing the financial impact of risk-sharing agreements and their potential to reduce treatment costs while maintaining patient access (14, 15). The budget impact analysis (BIA) complement cost-effectiveness analysis (CEA) by evaluating the affordability and financial impact of adopting new health technologies within a specific budget (16). Sensitivity and scenario analysis strengthens BIAs by assessing financial outcomes and implementation strategies in dynamic treatment landscapes with evolving therapies and uptake patterns (17). Stratified perspectives help assess financial feasibility and opportunity costs of implementation strategies (18). Understanding of the net budget impact is critical for healthcare planning and budget forecasting (19).

This paper focuses on health economics and outcome research, with particular emphasis on BIA of RRMS therapies. Although BIAs for RRMS have been conducted in other countries, comprehensive, nationally representative analyses incorporating the potential impact of MEAs are lacking in SA. This study provides an exploratory evaluation of the cost implications, affected patient populations, and managed entry strategies across different healthcare settings.

## 2 Methodology

### 2.1 Budget impact model structure and perspective

A static budget impact model was developed in Microsoft Excel, following the International Society for Pharmacoeconomics and Outcomes Research (ISPOR) BIA Good Practice II Guidelines (2014). It estimated the 5-year financial impact of managing MS in the adult Saudi national population from the Saudi Ministry of Health (MoH) perspective, with costs reported in Saudi Arabian Riyals (SAR). The model focused on treatment-eligible patients with RRMS, stratified into subgroups based on disease activity and treatment history-active RRMS naïve, highly active RRMS naïve, highly active RRMS non-naïve, aggressive RRMS naïve, and aggressive RRMS non-naïve that enabled detailed analysis of budgetary implications across varying severities (Supplementary Table S1). The model compared scenarios with and without intervention, analyzing differences in market share, resource use, and total costs. It included cost components such as drug acquisition, administration, monitoring, relapse, adverse events (AEs), hospitalization, and disease management. The primary outcome was the net budget impact, reflecting the cost difference between the two scenarios.

All drug acquisition costs in this analysis were sourced from the National Unified Procurement Company (NUPCO) pricing database as of May 2024. Healthcare resource utilization (HCRU) unit costs, including monitoring, relapse management, and AE management, were extracted from the MoH service code list. These unit costs are confidential but were reviewed and validated by subject matter experts within MoH to ensure accuracy and relevance to current clinical practice.

To inform epidemiological assumptions and treatment uptake for this study insights were gathered from key opinion leaders across the SA through structured primary market research interviews and Delphi panel rounds. In total, nine experts were involved at different stages during this analysis. The expert group included senior neurology consultants, academic researchers, and health policy advisors with direct involvement in national registries and value-based healthcare initiatives. Members of the panel hold senior appointments in national and regional health authorities, contributing rich field expertise. Many are affiliated with leading universities and possess a robust portfolio of publications in public health and related disciplines. Their contributions ensured regional relevance and methodological robustness in estimating disease prevalence and treatment adoption.

### 2.2 Model input

#### 2.2.1 Interventions

The model included a set of DMTs assigned to each RRMS subgroup based on disease activity and treatment history (Table 2). The comparator in this analysis was the standard of care (SOC) which comprised the DMTs currently available and used for the management of RRMS in SA. These included interferon-based therapies (interferon beta-1a, interferon beta-1b, interferon beta-1a), as well as glatiramer acetate formulations, dimethyl fumarate, teriflunomide, fingolimod and rituximab. For this analysis, patients were assumed to be on fixed-course therapy (e.g., cladribine) and were ineligible for other DMT for the entire study period. Patients who were on cladribine in year 1 were also assumed to be not on any other DMT during the study (as per the cladribine European Medicines Agency [EMA] label, following completion of the 2 treatment courses, no further cladribine treatment is required in years 3 and 4. Re-initiation of therapy after year 4 has not been studied). The switching between drugs was not incorporated in the model, however the budget impact was available by different population type.

**Table 2:** Interventions considered for each RRMS subgroup.

Active RRMS naïve	Highly active RRMS naïve	Highly active RRMS non-naïve	Aggressive RRMS naïve	Aggressive RRMS non-naïve
Peginterferon beta-1a	Cladribine	Cladribine	Natalizumab	Natalizumab
Siponimod	Natalizumab	Natalizumab	Ofatumumab	Ofatumumab
Ozanimod	Ofatumumab	Ofatumumab	Ocrelizumab	Ocrelizumab
Ofatumumab	Ocrelizumab	Ocrelizumab	–	–
Natalizumab	–	–	–	–
Ocrelizumab	–	–	–	–

‘–‘refers that the drug has not been included in a specific subgroup.

#### 2.2.2 Population

The target Saudi national population included eligible adult patients diagnosed with RRMS, stratified by treatment status (treatment-naïve or previously treated), disease severity (active or highly active RRMS), and disability status. The affected patient population was entered into the model based on the disease incidence and prevalence per year (20–23) (Supplementary Table S2). Epidemiological data was derived from expert consensus, statistical government reports (22, 24) and literature (20, 23).

#### 2.2.3 Drug costs

All DMTs currently reimbursed for RRMS in SA were included in the treatment modalities. The annual cost for each therapy spanned components such as drug acquisition costs, premedication and drug administration costs, monitoring costs, and total costs guided by local clinical practice; and costs associated with relapses, disease progression and AEs (Supplementary Tables S3–S5).

#### 2.2.4 Market share

Two market share scenarios were evaluated in the analysis. In the expert’s market share scenario, drug distribution was determined based on proportions estimated by clinical experts for each RRMS subgroup. In contrast, the 100% uptake scenario assumed that the entire eligible patient population within a given subgroup was treated with a single intervention. Both scenarios were analyzed with MEA and without MEA conditions to capture the effect of negotiated discounts on overall costs. For ocrelizumab, 3 distinct MEAs were applied, which were based on the total uptake (Tables 3, 4). In this analysis, MEAs were modeled as fixed percentage discounts on drug acquisition costs to reflect potential pricing structures under negotiated reimbursement agreements.

**Table 3:** Comparison of the MEAs for the key drugs evaluated in the study.

Drug	Eligible population	Assessment criteria	Duration
Cladribine	Naïve and non-naïve patients with highly active RRMS	Relapse (EDSS score) and MRI activity	5 years, renewed annually
Ozanimod	Adult patients with RRMS with active disease	Response at 3 and 6 months	3 years
Tysabri SC	All enrolled patients	Non-responders eligible for rebates	Not specified
Ofatumumab	Naïve and switched RRMS patients	Relapse and MRI findings	5 years
Ocrelizumab	Existing and new RRMS patients	Performance-based rebates	5 years

MEA, managed entry agreement; RRMS, relapsing-remitting multiple sclerosis; EDSS, Expanded Disability Status Scale; MRI, magnetic resonance imaging.

**Table 4:** The distinct MEA scenarios (ocrelizumab).

Scenario	Existing patients	New patients (MoH vs Sponsor)	Key features	Duration
MEA 1	100 patients (2 + 2) and 300 patients (3 + 1)	50 MoH vs. 40 Sponsor per year	Balanced uptake with moderate rebate structure	5 years
MEA 2	100 patients (2 + 2) and 300 patients (3 + 1)	70 MoH vs. 60 Sponsor per year	Increased MoH uptake with enhanced rebate	5 years
MEA 3	400 patients (3 + 1)	100 MoH vs. 120 Sponsor per year	High-volume uptake with maximum rebate potential	5 years

MEA, managed entry agreement; MoH, Ministry of Health.

### 2.3 Model output

The model estimated the net budget impact from the payer’s perspective in SA. Outputs were stratified by RRMS subtypes (active, highly active, and aggressive), and scenarios were analyzed both with and without MEA to capture the effect of the cost of all factors of each scenario, encompassing both individual and combined interventions upon overall expenditure.

### 2.4 Scenario analysis

The key cost drivers were identified with scenario analyses that identified the variations in key parameters, including distribution of therapeutic regimens, variations in population size, and application of MEA. The scenarios featured peginterferon beta-1a, siponimod, ozanimod, natalizumab, ofatumumab and ocrelizumab, and a combined scenario where all these drugs were considered together.

The combined scenario encompassed multiple RRMS subgroups and treatment options. For Active RRMS naïve patients, the therapies included peginterferon beta-1a, siponimod, ozanimod, ofatumumab, natalizumab, and ocrelizumab. In the highly active RRMS Naïve group and the highly active RRMS non-naïve groups, cladribine (Mavenclad®), natalizumab, ofatumumab, and ocrelizumab were considered. For both aggressive RRMS naïve and aggressive RRMS non-naïve patients, the treatments comprised natalizumab, ofatumumab, and ocrelizumab.

### 2.5 Sensitivity analyses

Sensitivity analysis was performed for the cladribine and ofatumumab-ocrelizumab combination scenarios to assess the robustness of model outcomes. These scenarios were selected due to their treatment paradigms and their potential to drive meaningful variability in model assumptions. The parameters included in the analyses were: (1) prevalence of multiple sclerosis; (2) percentage of RRMS patients within the total MS population; (3) proportion of RRMS patients eligible for treatment; (4) proportion of active RRMS patients; (5) proportion of highly active RRMS patients; (6) proportion of aggressive RRMS patients; and (7) proportion of treatment-naïve patients within these subgroups.

## 3 Results

**Figure 1:**
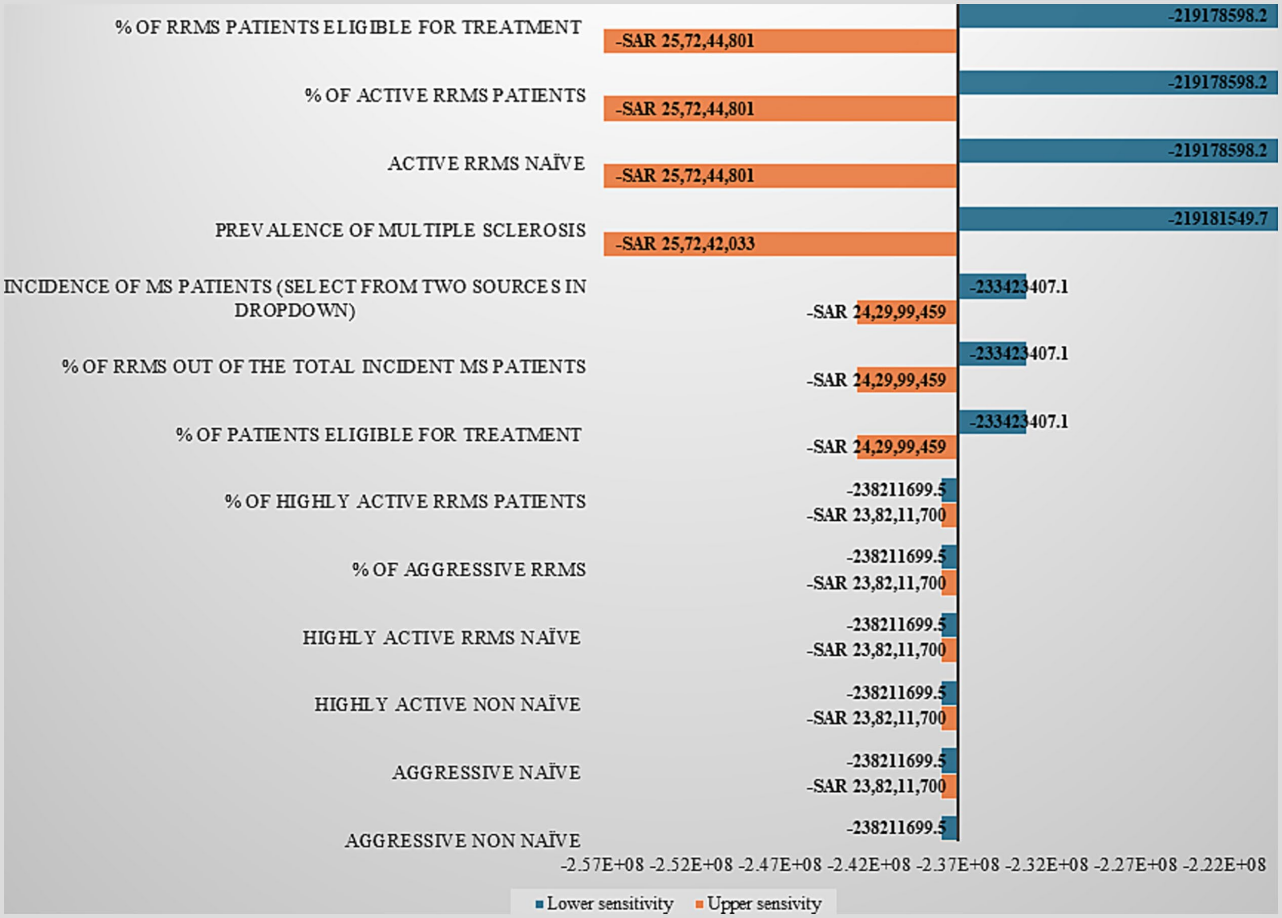
Sensitivity analysis for cladribine showing key drivers of model outcomes: tornado graph.

The results of one-way sensitivity analyses are presented in a tornado diagram which illustrates the relative influence of each variable on the total estimated cost. Sensitivity analyses of cladribine (Figure 1) highlight the key parameters influencing model outcomes related to MS prevalence, patient segmentation, and treatment eligibility. The model is most sensitive to assumptions on RRMS prevalence, treatment eligibility, and overall MS prevalence, while incidence-related factors show moderate influence. Sub-segmentation by disease activity or treatment history has minimal effect, indicating that accurate prevalence and eligibility inputs are critical for robust market and budget impact estimates.

**Figure 2:**
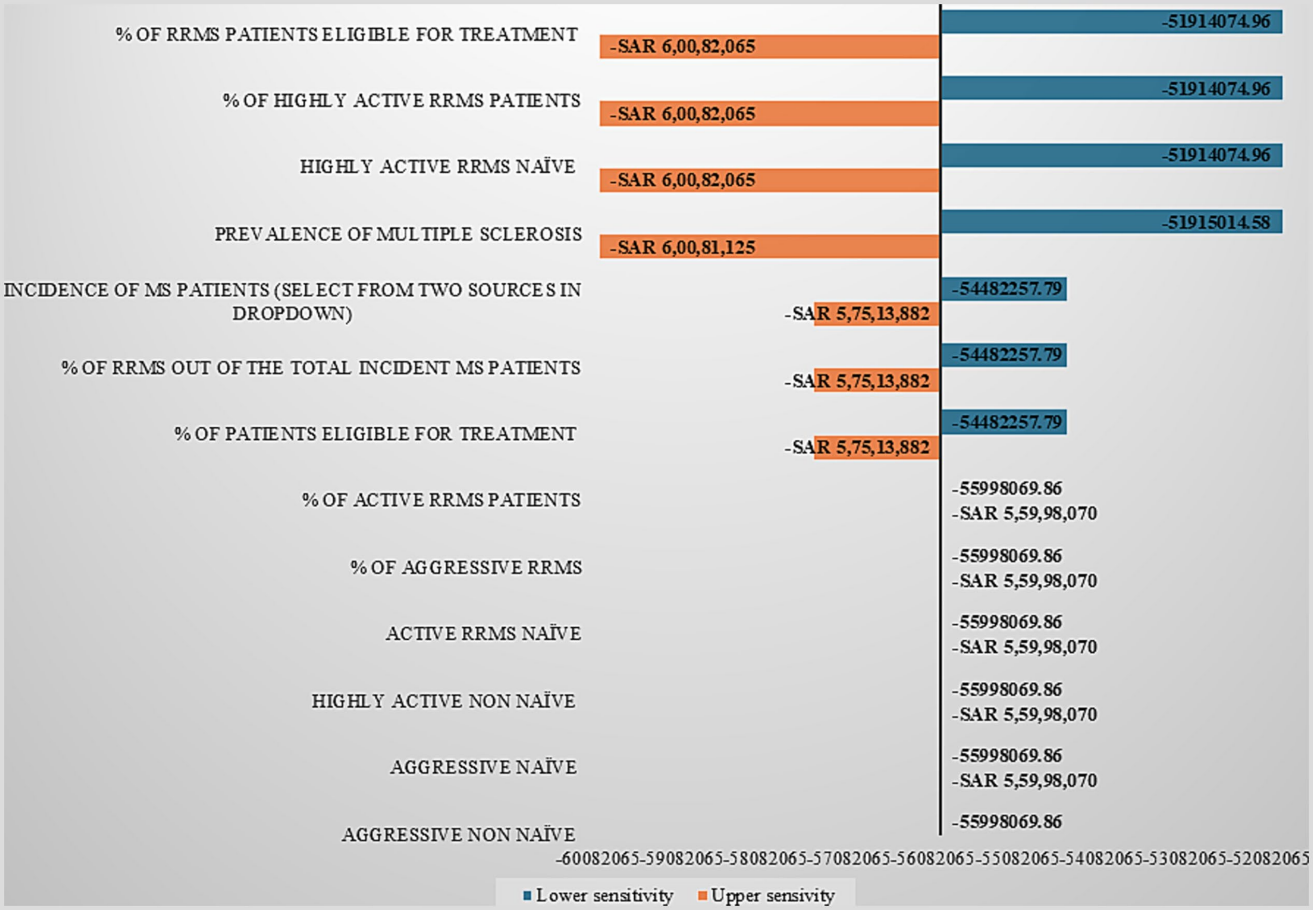
Sensitivity analysis for ofatumumab-ocrelizumab combination illustrating main model sensitivity drivers: tornado graph.

While analyses of combination of ofatumumab and ocrelizumab (Figure 2) indicate that model outcomes are chiefly influenced by parameters defining MS prevalence and treatment eligibility within the RRMS and highly active RRMS populations. Key sensitive drivers include the proportion of RRMS among total MS cases, RRMS treatment eligibility, prevalence rates, and the share of highly active RRMS patients. Incidence-related parameters demonstrated moderate influence, while further stratification by activity or treatment history had minimal impact. Overall, model sensitivity is primarily driven by prevalence- and eligibility-based assumptions, underscoring the importance of accurate epidemiological data to enhance model robustness and forecast reliability.

### 3.1 Active RRMS: naïve

Across all scenarios ocrelizumab was evaluated with three distinct MEAs based on patient location (Table 4). Under these agreements, it demonstrated notably lower acquisition costs compared with other therapies (Table 5). Nevertheless, in some scenarios, higher drug acquisition expenses were often balanced by a decrease in administration, monitoring, AEs and relapse costs. Care of relapses costs were reduced in all scenarios, thereby offsetting the overall expenditure to some extent.

**Table 5:** Budget impact of introducing new interventions for active RRMS naïve with and without MEA based on expert’s market share.

Scenario	Market share	With and without MEAs	Drug acquisition	Administration	Monitoring	Adverse events cost	Relapse cost	Total
Peginterferon beta-1a	1%	–	688,489.70	−87,388.58	18,370.64	−113,271.78	−3,080.41	503,119.56
Siponimod	3.75%	–	29,300,307.45	−468,589.52	484,297.03	86,845.44	−140,884.02	29,261,976.38
Ozanimod	1%	No MEA	2,426,343.42	−115,342.27	46,267.31	−39,001.99	−31,644.40	2,286,622.08
		MEA	1,610,109.87	−115,342.27	46,267.31	−39,001.99	−31,644.40	1,470,388.53
Natalizumab	10–12.5%	No MEA	44,476,407.59	−1,279,523.91	−258,668.39	−1,620,947.19	−1,205,014.90	40,112,253.19
		MEA	36,361,415.90	−1,279,523.91	−258,668.39	−1,620,947.19	−1,205,014.90	31,997,261.50
Ofatumumab	22–30%	No MEA	56,144,061.83	−3,284,776.73	690,425.12	855,196.85	−2,468,556.18	51,936,350.90
		MEA	16,470,698.69	−3,284,776.73	690,425.12	855,196.85	−2,468,556.18	12,262,987.76
Ocrelizumab	23–27%	No MEA	53,455,605.98	550,656.16	−555,669.98	852,028.83	−1,055,312.79	53,247,308.20
		MEA	−10,769,514.12	550,656.16	−555,669.98	852,028.83	−1,055,312.79	−10,977,811.90
Combined	–	No MEA	2,472,768,851.03	−3,439,242.42	90,188.22	269,926.06	−5,986,023.36	238,211,699.53
		MEA	133,514,359.77	−3,439,242.42	90,188.22	269,926.06	−5,986,023.36	124,449,208.27

MEA, managed entry agreement; Color red, orange, yellow, and green depict low to high costs.

*The values in the table are the difference in total cost from the standard of care (SOC) cost (Values of SOC: drug acquisition: 398,968,393.88; administration: 12,495,336.47; monitoring: 18,293,930.82; adverse events: 17,920,887.05; relapse cost: 15,599,633.66; total: 463,278,181.87).

Based on the expert’s market share, the implementation of MEAs led to moderate cost savings across multiple therapies. For instance, ocrelizumab’s total cost decreased by 2.37% after employing MEA. Drug acquisition costs were the primary cost drivers across most scenarios, particularly in the absence of MEA. Peginterferon beta-1a and siponimod, although not part of MEA scenarios, had a slight rise in cost by 0.1 and 6.3%, respectively.

In 100% uptake scenarios, costs were significantly higher without MEA. Siponimod was multifold more expensive than peginterferon beta-1a at full uptake. Ocrelizumab with MEA (three price variants) ranged between 69.48–73.98% which was consistently lower than its non-MEA counterpart. Across all scenarios analyzed, drug acquisition cost emerged as the primary driver of total expenditure in treating RRMS, with other components including administration, monitoring, AEs, and relapse management contributing to a lesser extent (Supplementary Table S6).

The maximum budget impact was observed in the siponimod (100% uptake) scenario, while the minimum was in the ocrelizumab (expert’s market share) with the MEA scenario.

### 3.2 Highly active RRMS: naïve

The introduction of these therapies for highly active RRMS in naïve patients demonstrated higher costs, primarily driven by increased drug acquisition expenses in most of the scenarios (Table 6). However, none of the scenarios showed an increase in drug administration and relapse care costs.

**Table 6:** Budget impact of introducing new interventions for highly active RRMS naïve with and without MEA based on expert’s market share.

Scenario	Market share	With and without MEA	Drug acquisition	Administration	Monitoring	Adverse events cost	Relapse cost	Total
Cladribine	5%	No MEA	6,682,502.09	−494,894.79	−36,502.47	−11,618.37	−273,427.08	5,866,059.37
		MEA	1,289,755.46	−494,894.79	−36,502.47	−11,618.37	−273,427.08	473,312.75
Natalizumab	20–23%	No MEA	13,654,897.17	−1,112,528.00	53,221.76	−789,694.69	−938,158.93	10,867,737.31
		MEA	10,285,917.64	−1,112,528.00	53,221.76	−789,694.69	−938,158.93	7,498,757.78
Ofatumumab	44–57%	No MEA	11,405,141.58	−2,647,610.94	646,547.86	83,248.10	−1,862,660.40	7,624,666.19
		MEA	−5,435,145.67	−2,647,610.94	646,547.86	83,248.10	−1,862,660.40	−9,215,621.06
Ocrelizumab	24–28%	No MEA	8,159,370.40	−104,276.12	−63,228.50	101,220.50	−342,186.26	7,750,900.01
		MEA	−5,166,697.83	−104,276.12	−63,228.50	101,220.50	−342,186.26	−5,575,168.21
Combined	–	No MEA	43,165,840.70	−3,506,466.15	335,977.76	−174,050.83	−3,117,399.89	36,703,901.59
		MEA	9,916,694.72	−3,506,466.15	335,977.76	−174,050.83	−3,117,399.89	3,454,755.62

MEA, managed entry agreement, Color red, orange, yellow, and green depict low to high costs.

*The values in the table are the difference in the total cost from the standard of care (SOC) cost. (Values of SOC: drug acquisition: 106,289,311.81; administration: 5,269,451.17; monitoring: 3,119,689.69; adverse events: 4,293,635.56; relapse cost: 6,088,125.18; total: 125,060,213.42).

Based on the expert’s market share analysis, the combined scenario showed the highest cost contribution without MEA, with an increase of 29.35%. Ofatumumab and ocrelizumab with MEA demonstrated the highest cost savings, lowering costs by 7.37 and 4.46%, respectively. The introduction of MEAs led to notable reductions in drug acquisition costs, particularly for ofatumumab and ocrelizumab, which drove overall savings. Additionally, reduced relapse and AE costs further contributed to cost savings in MEA scenarios.

For 100% uptake, cladribine and ofatumumab with MEA cut costs by 17.66 and 12.97%, making them net savings, with all scenarios showing lower administration and relapse care costs (Supplementary Table S7).

The maximum budget impact was for ocrelizumab at 100% uptake without MEA, driven by high drug and AE costs, while the minimum was for cladribine at 100% uptake with MEA, reflecting major savings from reduced drug acquisition, relapse, and AE expenses.

### 3.3 Highly active RRMS: non-naïve

The rise in acquisition costs resulted in higher overall expenses in most of the scenarios, but savings from other healthcare components helped reduce the financial impact (Table 7).

**Table 7:** Budget impact of introducing new interventions for highly active RRMS non naïve with and without MEA based on expert’s market share.

Scenario	Market share	With and without MEA	Drug acquisition	Administration	Monitoring	Adverse events cost	Relapse cost	Total
Cladribine	5%	No MEA	1,650,443.15	−152,194.86	−18,939.09	−3,572.99	−84,086.96	1,391,649.25
		MEA	122,949.11	−152,194.86	−18,939.09	−3,572.99	−84,086.96	−135,844.79
Natalizumab	20–23%	No MEA	4,326,973.53	−352,538.66	16,864.95	−250,239.02	−297,284.47	3,443,776.33
		MEA	3,270,271.27	−352,538.66	16,864.95	−250,239.02	−297,284.47	2,387,074.07
Ofatumumab	44–57%	No MEA	3,595,129.58	−834,580.12	203,804.86	26,241.47	−587,147.95	2,403,447.84
		MEA	−2,445,411.14	−834,580.12	203,804.86	26,241.47	−587,147.95	−3,637,092.87
Ocrelizumab	24–28%	No MEA	2,535,467.79	−32,860.88	−19,388.25	31,661.90	−106,740.36	2,408,140.19
		MEA	−2,350,447.05	−32,860.88	−19,388.25	31,661.90	−106,740.36	−2,477,774.65
Combined	–	No MEA	13,120,299.89	−1,102,681.00	99,228.46	−56,095.45	−980,182.34	11,080,569.56
		MEA	1,354,976.55	−1,102,681.00	99,228.46	−56,095.45	−980,182.34	−684,753.78

MEA, managed entry agreement; Color red, orange, yellow, and green depict low to high costs.

*The values in the table are the difference in the total cost from the standard of care (SOC) cost (Values of SOC: drug acquisition: 33,885,730.35; administration: 1,679,935.63; monitoring: 994,577.55; adverse events: 1,368,839.20; relapse cost: 1,940,934.27; total: 39,870,017.01).

With MEA implementation, costs decreased for all therapies except natalizumab, including reductions of 0.34% for cladribine, 9.12% for ofatumumab, 6.22% for ocrelizumab, and 1.72% in the combined scenario, based on the expert’s market share analysis. Natalizumab was the only therapy that did not achieve overall cost savings, as its drug acquisition cost remained relatively high even with MEA application.

Upon 100% uptake, all therapies showed relapse-related cost savings, with natalizumab and ocrelizumab providing the greatest reductions. The most significant driver across all scenarios was drug acquisition cost, with ocrelizumab and natalizumab incurring the highest acquisition costs without MEA. However, administration, monitoring, AE, and relapse costs also contribute to overall differences. MEA contracts significantly reduce acquisition costs, particularly benefiting high-cost drugs like cladribine and ofatumumab (Supplementary Table S8).

Across scenarios, the highest budget impact was observed for ocrelizumab without MEA at 100% uptake (SAR 26,329,248), while the lowest and cost-saving scenario was cladribine with MEA at 100% uptake (SAR –10,373,380).

### 3.4 Aggressive RRMS: naïve

As per experts’ market share, MEAs led to significant budget relief in aggressive disease treatment in treatment-naïve patients (Table 8). With MEA introduction, total costs declined by 6.23% for ofatumumab, 5.81% for ocrelizumab, and 7.63% in the combined scenario. These cost savings were primarily driven by notable reductions in drug acquisition costs, which constitute the largest proportion of total expenses.

**Table 8:** Budget impact of introducing new interventions for aggressive RRMS naïve with and without MEA based on expert’s market share.

Scenario	Market share	With and without MEAs	Drug acquisition	Administration	Monitoring	Adverse events cost	Relapse cost	Total
Natalizumab	5–13%	No MEA	2,587,537.99	−226,964.13	25,711.90	−161,186.66	−215,451.29	2,009,647.82
		MEA	1,899,189.66	−226,964.13	25,711.90	−161,186.66	−215,451.29	1,321,299.49
Ofatumumab	27–30%	No MEA	2,642,311.95	−716,539.07	216,243.56	7,559.16	−572,078.95	1,577,496.66
		MEA	−2,645,741.27	−716,539.07	216,243.56	7,559.16	−572,078.95	−3,710,556.56
Ocrelizumab	25–29%	No MEA	3,166,567.38	−56,319.85	−1,354.51	36,373.03	−173,363.86	2,971,902.19
		MEA	−3,271,736.18	−56,319.85	−1,354.51	36,373.03	−173,363.86	−3,466,401.36
Combined	–	No MEA	9,781,271.92	−1,150,213.92	215,635.30	34,931.90	−1,090,277.36	7,791,347.84
		MEA	−2,554,939.92	−1,150,213.92	215,635.30	34,931.90	−1,090,277.36	−4,544,864.00

MEA, managed entry agreement; Color red, orange, yellow, and green depict low to high costs.

*The values in the table are the difference in the total cost from the standard of care (SOC) cost (Values of SOC: drug acquisition: 50,550,869.74; administration: 2,513,385.91; monitoring: 1,285,111.15; adverse events: 2,041,522.98; relapse cost: 3,227,833.60; total: 59,618,723.37).

In the 100% uptake scenario, ofatumumab with MEA reduced costs by 16.2%, likely driven by significant reductions in drug acquisition and relapse-related costs. Drug administration and relapse care costs consistently decreased across all scenarios, contributing significantly to overall cost savings (Supplementary Table S9).

At 100% uptake, the greatest budget impact is seen with ocrelizumab without an MEA (SAR 36,780,335), while the lowest and lowest cost-saving scenario is ofatumumab with an MEA (SAR -9,654,898).

### 3.5 Aggressive RRMS non-naïve

Table 9 outlines the aggressive RRMS non-naïve scenarios under both MEA and non-MEA conditions. Under expert-based assumptions, ofatumumab, ocrelizumab and combined scenario showed a decline in total cost by 19.5, 7.29 and 22.31%, respectively. This reflects a marked financial reversal, suggesting potential cost savings or returns that outweighed the initial expenditure.

**Table 9:** Budget impact of introducing new interventions for aggressive RRMS non-naïve with and without MEA based on expert’s market share.

Scenario	Market share	With and without MEAs	Drug acquisition	Administration	Monitoring	Adverse events cost	Relapse cost	Total
Natalizumab	5–13%	No MEA	343,806.92	−30,156.79	3,416.35	−21,416.92	−28,627.07	267,022.49
		MEA	253,817.38	−30,156.79	3,416.35	−21,416.92	−28,627.07	177,032.94
Ofatumumab	27–30%	No MEA	362,006.48	−98,168.50	29,626.17	1,035.63	−78,376.93	216,122.86
		MEA	−1,452,975.09	−98,168.50	29,626.17	1,035.63	−78,376.93	−1,598,858.71
Ocrelizumab	25–29%	No MEA	424,372.80	−7,597.31	−188.17	4,921.88	−23,255.95	398,253.26
		MEA	−570,780.20	−7,597.31	−188.17	4,921.88	−23,255.95	−596,899.75
Combined		No MEA	1,328,852.78	−155,537.83	29,453.57	4,678.56	−147,904.75	1,059,542.34
		MEA	−1,559,115.66	−155,537.83	29,453.57	4,678.56	−147,904.75	−1,828,426.10

MEA, managed entry agreement; Color red, orange, yellow, and green depict low to high costs.

*The values in the table are the difference in the total cost from the standard of care (SOC) cost (Values of SOC: drug acquisition: 6,950,105.01; administration: 345,558.76; monitoring: 176,686.52; adverse events: 280,683.58; relapse cost: 443,786.28; total: 8,196,820.16).

With 100% uptake, ofatumumab achieved the highest cost savings with a 29% decline, primarily driven by reductions in drug acquisition, administration, and relapse costs. Most therapies showed reduced relapse costs, contributing positively to overall savings.

The highest budget impact was for ocrelizumab (100% uptake, without MEA), while the lowest was for ofatumumab (with MEA) (Supplementary Table S10).

### 3.6 Ofatumumab and ocrelizumab combined

The introduction of ofatumumab and ocrelizumab across different RRMS populations, including both naïve and non-naïve, resulted in a notable shift in cost dynamics, which was driven by elevated drug acquisition costs (Table 10). In the prevalent RRMS population, the market share was predominantly held by ofatumumab, which accounted for 81% of usage, whereas ocrelizumab occupied the remaining 19%.

**Table 10:** Budget impact of introducing both ofatumumab and ocrelizumab with and without MEA based on market share split.

Budget impact	Market share	Drug acquisition	Administration	Monitoring	Adverse events cost	Relapse cost	Total
Active RRMS naïve							
Without MEA	Ocrelizumab is 16.5% & Ofatumumab 83.5%	222,025,725.61	−9,198,909.33	1,297,318.27	4,178,550.62	−214,347.08	218,088,338.09
With MEA 1		167,923,580.95	−9,198,909.33	1,297,318.27	4,178,550.62	−214,347.08	163,986,193.43
With MEA 2		165,270,273.39	−9,198,909.33	1,297,318.27	4,178,550.62	−214,347.08	161,332,885.86
With MEA 3		158,444,890.69	−9,198,909.33	1,297,318.27	4,178,550.62	214,347.08	154,507,503.16
Highly Active RRMS Naïve							
Without MEA	Ocrelizumab is 19% & Ofatumumab 81%	29,193,322.15	−4,467,266.72	936,573.81	391,610.25	702,150.74	26,756,390.23
With MEA 1		17,184,728.12	−4,467,266.72	936,573.81	391,610.25	702,150.74	14,747,796.20
With MEA 2		16,668,807.21	−4,467,266.72	936,573.81	391,610.25	702,150.74	14,231,875.29
With MEA 3		15,341,649.46	−4,467,266.72	936,573.81	391,610.25	702,150.74	12,904,717.54
Highly Active RRMS non-naïve							
Without MEA	Ocrelizumab is 19% & Ofatumumab 81%	8,895,621.48	−1,448,102.16	309,350.37	118,088.59	222,217.42	8,097,175.70
With MEA 1		3,955,308.32	−1,448,102.16	309,350.37	118,088.59	222,217.42	3,156,862.54
With MEA 2		3,734,199.36	−1,448,102.16	309,350.37	118,088.59	222,217.42	2,935,753.58
With MEA 3		3,165,417.47	−1,448,102.16	309,350.37	118,088.59	222,217.42	2,366,971.69
Aggressive RRMS naïve							
Without MEA	Ocrelizumab is 21% & Ofatumumab 79%	16,135,397.90	−2,112,744.56	578,331.21	139,398.44	330,715.97	15,071,098.95
With MEA 1		9,754,537.57	−2,112,744.56	578,331.21	139,398.44	330,715.97	8,690,238.61
With MEA 2		9,503,947.41	−2,112,744.56	578,331.21	139,398.44	330,715.97	8,439,648.45
With MEA 3		8,859,327.93	−2,112,744.56	578,331.21	139,398.44	330,715.97	7,795,028.98
Aggressive RRMS non-naïve							
Without MEA	Ocrelizumab is 21% & Ofatumumab 79%	2,150,399.38	−294,427.92	81,292.61	18,048.13	45,170.74	2,000,482.94
With MEA 1		134,955.21	−294,427.92	81,292.61	18,048.13	45,170.74	−14,961.23
With MEA 2		90,733.41	−294,427.92	81,292.61	18,048.13	45,170.74	−59,183.02
With MEA 3		−23,022.97	−294,427.92	81,292.61	18,048.13	45,170.74	−172,939.40

MEA, managed entry agreement. Color red, orange, yellow, and green depict low to high costs.

*The values in the table are the difference in the total cost from the standard of care (SOC) cost (*SOC for active RRMS naïve:* drug acquisition: 446,821,638.90; administration: 12,495,328.34; monitoring: 18,141,832.42; adverse events: 17,920,875.40; relapse cost: 8,906,205.83; total: 504,285,880.90; *SOC for highly active RRMS naïve:* drug acquisition: 113,539,980.94; administration: 5,269,453.46; monitoring: 3,109,311.38; adverse events: 4,293,637.43; relapse cost: 2,635,938.89; Total: 128,848,322.09; *SOC for highly active RRMS non naïve:* drug acquisition: 36,197,016.76; administration: 1,679,923.61; monitoring: 991,261.36; adverse events: 1,368,829.42; relapse cost: 840,348.25; total: 41,077,379.40; *SOC for aggressive RRMS naïve*: drug acquisition: 50,550,838.97; administration: 2,513,384.38; monitoring: 1,285,110.36; adverse events: 2,041,521.73; relapse cost: 1,383,557.20; total: 57,774,412.65; *SOC for aggressive RRMS non naïve*: drug acquisition: 6,950,055.32; administration: 345,556.29; monitoring: 176,685.26; adverse events: 280,681.57; relapse cost: 190,220.37; total: 7,943,198.81).

In most of the indications, there was a significant rise in drug acquisition costs over the 5-year time horizon upon utilization of dual drugs. However, this was partially offset by reductions in administration costs and a decline in relapse care costs. Overall, while administration, monitoring, and relapse-related costs remained relatively stable, the inclusion of MEAs substantially mitigated the drug acquisition burden across all disease severities and treatment.

Nevertheless, a decrease in drug acquisition costs was observed exclusively in scenarios involving MEA. All MEAs in aggressive RRMS non-naïve demonstrated a decrease in drug acquisition costs in contrast to all other scenarios.

The highest total budget impact occurred in the active RRMS naïve population without MEA, while the lowest was in the aggressive RRMS non-naïve population under MEA 3, resulting in a net cost-saving scenario driven by markedly lower drug acquisition and administration costs. Table 8 summarizes the ofatumumab and ocrelizumab combined scenarios reflecting market share split for both MEA and non-MEA settings.

## 4 Discussion

The comprehensive approach for BIA adopted in this study provided a detailed understanding of the net budget impact associated with each treatment strategy.

Data from real-world settings reported the effectiveness of peginterferon beta-1a (25), siponimod (26), ozanimod (27), natalizumab (28, 29), ofatumumab (30, 31), ocrelizumab (32, 33), and cladribine (34, 35) for RRMS therapy due to their widespread use as high-efficacy DMTs across varying disease severities and patient populations. MEAs have emerged as a key mechanism for mitigating budgetary pressures, offering financial relief through negotiated discounts, rebates, and outcome-based arrangements. MEA reduce risks from suboptimal coverage and promote a flexible, patient-centered healthcare system (36).

### 4.1 Impact of MEA’S on the budget

The implementation of MEAs led to salient cost savings across multiple therapies under both expert-estimated and full uptake scenarios. Siponimod’s high budget is predominantly driven by substantial drug acquisition expenses, with minimal offsets from administration and relapse cost savings. In treatment-naïve RRMS patients, ocrelizumab, ozanimod, natalizumab, and ofatumumab showed cost reduction to varying degrees. Highly active RRMS naïve demonstrated cost savings, suggesting larger rebates or outcome-based offsets. Cladribine demonstrated approximately 18% decrease in costs, indicating larger rebates and outcome-based offsets. The BIA analysis conducted by Bohlega et al. demonstrated that cladribine was associated with significant cost savings over a five-year period in SA. This primarily driven by drug administration and AE management costs (37). In previously treated highly active RRMS patients, under expert uptake, all the scenarios demonstrated a decrease in cost with MEA. It also led to considerable decrease in budget in aggressive diseases in treatment-naïve and non-naïve patients. Under MEA, a reduction in costs by 4–8% was observed across different scenarios. Ocrelizumab and natalizumab showed substantial cost savings. Ocrelizumab was found to be cost-effective versus natalizumab in Italy (38) and reduced the RRMS budget by 4.82% over 5 years in Costa Rica (39). The MEA transformed the treatment cost from a financial burden to a surplus, demonstrating its strong potential for cost containment and value generation.

### 4.2 Effect of 100% uptake

Scaling the model to full uptake revealed substantial cost differentials, indicating a marked escalation in the overall economic burden with broader patient coverage. In this BIA, the 100% uptake scenario represents a theoretical maximum in which all eligible patients received DMTs. Recent CEAs found ofatumumab to be cost-effective for RRMS treatment in both SA and Greece (40, 41). Ocrelizumab MEAs ensured stable and predictable cost containment, with evidence from Portugal supporting its cost-saving potential in active RRMS (42). In non-naïve highly active RRMS, all DMTs showed relapse-related cost savings, with natalizumab and ocrelizumab offering the greatest reductions. Ocrelizumab’s consistent savings suggest its effectiveness stems from clinical performance evident from real-world settings (43). Full uptake scenarios in aggressive RRMS (naïve and non-naïve) highly burdensome subgroup highlight the significant budget implications of high-efficacy DMTs. All scenarios of ocrelizumab, natalizumab and ofatumumab proved to be cost-effective compared to SOC’s. The CEAs from Brazil reported natalizumab to be cost-effective for highly active RRMS from both public and private healthcare perspectives (44, 45). In the 100% uptake scenario, where all eligible patients used DMTs, overall costs were lower.

### 4.3 Drug-specific cost dynamics

The analysis of drug-specific cost dynamics revealed noteworthy variations across therapies, highlighting differences in economic impact. In active RRMS naïve, high-efficacy therapies such as ocrelizumab and ofatumumab demonstrated substantially reduced relapses and AE costs. But they were associated with higher acquisition costs. Ofatumumab significantly reduces disability progression and is cost-effective for treating RRMS in SA (40). This indicated superior clinical performance that may offset its higher upfront costs (46). Ocrelizumab under various MEA models maintains consistent savings, suggesting pricing predictability and controlled economic outcomes despite variability in administration and acquisition inputs. The drugs are also presented as the most favorable in terms of relapse and AE cost containment, further supporting their therapeutic effectiveness in highly active RRMS naïve cases. A study showed the cost-effectiveness of ocrelizumab for relapse cases in Portugal, but with PPMS (47). Ofatumumab also presents a compelling case for MEA application as its high total cost transitioned to a budget-saving scenario under MEA, reflecting the efficacy of deep rebate mechanisms. Natalizumab, a clinically robust DMT, in comparison to other drugs and SOC, is burdened by substantial acquisition, culminating in high total cost even under expert uptake with MEA. Though the BIA analysis from US showed it is cost-effective due to cumulative savings for over 3 years increasing annual per-patient savings (48). For non-naïve cases, cladribine continues to demonstrate strong economic favorability, resulting in notable budget savings under both expert and full uptake scenarios when supported by MEA. Existing literature suggests cladribine to be a cost-effective and economically dominant treatment for RRMS, offering better quality of life at lower costs and demonstrating significant budget savings over 5 years (49). Nevertheless, natalizumab is associated with low relapse and AE costs, but due to its high drug acquisition cost, it was found to be economically intensive therapy in both uptake conditions for such patients. Conversely, both ocrelizumab and ofatumumab show favorable cost–benefit profiles with MEA, primarily due to their more manageable relapse-associated expenditures. Chiao and Meyer showed that despite higher acquisition costs, natalizumab was the most cost-effective therapy per relapse avoided, with minimal impact on health-plan costs (50). For aggressive naïve cases, ocrelizumab again demonstrated the optimal balance of efficacy and economic sustainability across all MEA. Ofatumumab, while initially incurring high acquisition and administration costs without MEA, benefits from its implementation. Natalizumab, although comparatively less expensive at acquisition, accumulates higher relapse-related expenses. A similar outcome was previously reported with natalizumab in patients with relapsing MS (50). Without MEA, ofatumumab represented the costliest option, but MEA application resulted in a decrease in cost under 100% uptake. Ocrelizumab also offered a balanced cost profile and showed substantial savings through MEA models. Natalizumab is challenged by high relapse costs, diminishing its overall economic appeal despite MEA support.

Overall, MEA application across RRMS subpopulations resulted in substantial cost savings. Ocrelizumab generally incurs higher baseline drug acquisition costs than ofatumumab but benefits significantly from MEA. Ocrelizumab, particularly in MEA 3 scenario, achieved the lowest total costs across multiple patient cohorts.

### 4.4 Drivers of cost

Across all RRMS subtypes and treatment settings, AE costs emerge as a significant contributor to overall economic burden. In highly active and active naïve RRMS populations, cladribine and natalizumab consistently demonstrated lower AE costs, reflecting their favorable safety profiles. The association of lower AE costs with cladribine is also reported (37). Ocrelizumab and ofatumumab maintained cost efficacy through lower AE incidence, despite higher acquisition costs. Though a country-wise shift of perspective reports ocrelizumab to be cost-effective for RRMS in the Iranian population (51) but not in the Colombian population (52). Within highly active and aggressive RRMS naïve and non-naïve populations, the AE costs remain unchanged for all therapies.

Further, relapse costs constitute a dominant component of total cost in nearly all subpopulations and treatment scenarios. These costs were consistently reduced for all therapies, with the greatest savings seen at 100% uptake. Cladribine was associated with higher savings than other drugs. This consistent trend highlights the effectiveness of these treatments in minimizing the economic burden associated with relapse-related costs. Cladribine and ofatumumab demonstrated net savings due to favorable pricing structures, clinical effectiveness, and minimal resource use.

This economic evaluation explored the DMTs regarding RRMS therapies and MEAs, but its reliance and assumptions may limit real-world applicability, especially given variability in healthcare access, adherence, and system-level factors in SA. Real-world evidence is essential to guide clinical and policy decisions. The inclusion of costly, innovative therapies also raises concerns about long-term healthcare sustainability, highlighting the need for value-based frameworks and outcome monitoring to support efficient resource allocation.

## 5 Limitations

Due to the limited availability of studies from SA or the wider Gulf Cooperation Council region, our findings were compared and contextualized using evidence from international studies. Moreover, real-world data on MS in SA remains limited and fragmented, with incomplete longitudinal follow-up and challenges related to patient mobility, particularly among expatriate populations. These data constraints influence the precision of model inputs and assumptions regarding treatment uptake and relapse outcomes.

This analysis was based on publicly available list prices, as confidential procurement and rebate data for DMTs in SA were unavailable. Consequently, the estimated budget impact may not fully represent real-world expenditures, since actual negotiated prices under MEAs or institutional contracts could differ substantially. Furthermore, the model did not account for regional variations in treatment access, adherence, or healthcare resource utilization, which may influence overall cost outcomes.

Additionally, MEA implementation in the region is still at an early stage, with most current agreements being financial rather than outcome-based. Effective adoption of such agreements requires robust real-world data infrastructure, continuous monitoring, and governance frameworks that may not yet be fully established. These factors are critical to interpret the magnitude of MEA-related savings and their implications for national affordability and formulary decision-making. Moreover, the analysis excluded indirect costs such as productivity losses and caregiver burden, which are substantial in MS and could further influence the overall economic impact. The kingdom’s mixed funding model allows access to high-cost therapies, specialist MS centers. MEA may also differ across sectors and population groups (for example, Saudi citizens vs. expatriates; public vs. private insurance status). Moreover, geographical disparities (remote or rural regions vs. major urban hubs) may further limit timely access to specialist care and follow-up required for performance-based MEA. As a result, while our budget impact and MEA modeling assume broad uptake and access, real-world implementation may be constrained by differential access and affordability, reducing generalizability of our findings across the entire RRMS population in SA.

Collectively, these factors are critical to interpret the magnitude of MEA-related savings and the generalizability of the findings to the broader RRMS population in SA.

## 6 Conclusion

This BIA underscores the critical role of MEAs in improving the affordability and sustainability of high-cost, high-efficacy therapies for RRMS. Across all scenarios, MEAs significantly reduce the overall treatment and relapse-related costs, particularly for ofatumumab and ocrelizumab. Cladribine consistently emerges as a cost-efficient option and may be strategically positioned as a first-line agent in appropriate patients. Conversely, natalizumab remains associated with higher acquisition cost and lower relapse and AE costs, likely due to its safety and effectiveness profile. Strategic prioritization of MEA discussions for ofatumumab and ocrelizumab, along with the inclusion of cladribine as a cost-effective first-line treatment option, may optimize resource allocation and improve access for eligible RRMS patients.

Real-world constraints such as limited data availability, differential access, and lack of confidential pricing may affect the accuracy and generalizability of the estimated budget impact. These factors are essential when interpreting MEA-related savings and their relevance to national formulary decision. Future efforts should focus on strengthening real-world data infrastructure and governance to support outcome-based MEAs and improve the precision of economic evaluations.

## Data Availability

The original contributions presented in the study are included in the article/Supplementary material, further inquiries can be directed to the corresponding author.
