# Correction: Balancing innovation and affordability in relapsing-remitting multiple sclerosis: a budget impact analysis from Saudi Arabia

**DOI:** 10.3389/fpubh.2025.1763073

**Published:** 2026-01-12

**Authors:** Ahmed Al-Jedai, Hajer Almudaiheem, Faisal A. Al-Suweidan, Mohammed Al Jumah, Ahmed al-Thobaiti, Fahad Alzureiqan, Wejdan Abu Ras, Fahad M. Al-Dosari, Yaser Al Malik, Pratik Dhopte, Rita Ojeil

**Affiliations:** 1 Colleges of Medicine and Pharmacy, Alfaisal University, Riyadh, Saudi Arabia; 2 The Saudi Society of Clinical Pharmacy, Riyadh, Saudi Arabia; 3 Division of Neurology, Department of Medicine, Security Forces Hospital, Riyadh, Saudi Arabia; 4 King Abdullah International Medical Research Center (KAIMRC), Riyadh, Saudi Arabia; 5 Department of Neurology, King Saud bin Abdulaziz University for Health Sciences, Riyadh, Saudi Arabia; 6 King Saud Medical City, Riyadh, Saudi Arabia; 7 Drug Policy & Regulation Unit, Ministry of Health, Riyadh, Saudi Arabia; 8 Therapeutic Affairs Deputyship, Saudi Ministry of Health, Riyadh, Saudi Arabia; 9 Department of Clinical Pharmacy, King Saud Medical City, Riyadh First Health Cluster (R1), Ministry of Health, Riyadh, Saudi Arabia; 10 College of Medicine, King Saud bin Abdulaziz University for Health Sciences, Riyadh, Saudi Arabia; 11 Department of Neurology, King Abdulaziz Medical City, National Guard Health Affairs, Riyadh, Saudi Arabia; 12 Carexso, Dubai, United Arab Emirates; 13 PDC-CRO, Dubai, United Arab Emirates

**Keywords:** budget impact analysis, relapsing-remitting multiple sclerosis, managed entry agreements, disease-modifying therapies, multiple sclerosis

Affiliation ^3^Division of Neurology, Department of Medicine, Security Forces Hospital, Riyadh, Saudi Arabia was erroneously given as ^3^Critical Care Medicine, Security Forces Hospital, Riyadh, Saudi Arabia.

The original version of this article has been updated.

