# Supplementary material for: Balancing innovation and affordability in relapsing-remitting multiple sclerosis: a budget impact analysis from Saudi Arabia
Source: Front Public Health. 2025 Dec 1;13:1713092. doi: 10.3389/fpubh.2025.1713092 (PMC12702914; doi:10.3389/fpubh.2025.1713092)
Supplement: Supplementary file 1 [file Data_Sheet_1.pdf]

Table S1. Details of Model Structure

Key Model Elements	
**Model Settings**	Inputs
Patient Population	The adult Saudi National population with MS are further categorized as people:<br>• With RRMS<br>• Who are eligible for treatment<br>• With high disease activity<br>• With aggressive RRMS
Analytical tool	Microsoft Excel
Model time horizon	5 years
Country	Saudi Arabia (KSA)
Currency	Saudi Arabi Riyal (SAR)
Perspective	Ministry of Health (MoH)
**Model Overview**	
**Model Component**	**Details**
**Model Type**	Static Budget Impact Model
**Population**	Treatment Eligible RRMS Patients
**RRMS Subgroups**	• Active RRMS / Highly Active RRMS<br>Aggressive RRMS
**Subgroup Breakdown**	• Active RRMS Naïve<br>• Highly Active RRMS Naïve<br>• Highly Active RRMS Non-Naïve<br>• Aggressive RRMS Naïve<br>• Aggressive RRMS Non-Naïve
**Model Entry**	• Eligible Target Patient Population Group Enters the Model
**Scenario Comparison**	Scenario Without Intervention:<br>• Market Share<br>• Resource Use<br>• Total Budget Costs<br>Scenario With Intervention:<br>• Market Share<br>• Resource Use<br>• Budget Costs
**Cost Components Considered**	• Drug Costs<br>• Administration Costs<br>• Monitoring Cost<br>• Relapse Cost

Key Model Elements	
	• Adverse Event Cost<br>• Management Cost<br>• Hospitalization Cost
**Outcome**	• Net Budget Impact = Difference between total budget costs in both scenarios (scenarios with intervention and scenarios without intervention)
**Uptake**	• Resource Use includes uptake of prescribed/new DMTs.

RRMS: Relapse Remitting Multiple Sclerosis

Table S2. Population Inputs

Disease	2024	2025	2026	2027	2028
Active RRMS naive	2,257	2,461	2,667	2,876	3,089
Highly Active RRMS naive	453	507	562	617	673
Highly Active RRMS non-naive	174	177	179	182	185
Aggressive RRMS naive	216	238	260	283	306
Aggressive RRMS non-naive	35	35	36	36	37

RRMS: Relapse Remitting Multiple Sclerosis
Source: KOI Insights; Etemadifar et al., 2020, Alsaedi et al., 2022; General Authority of Statistics 2021, World Bank

Table S3: Drug costs summary

Treatment	Dose	Drug acquisition costs (SAR)		Administration costs (SAR)		Monitoring costs (SAR)		Total costs (SAR)	
		Year 1	Year 2+ onwards	Year 1	Year 2+ onwards	Year 1	Year 2+ onwards	Year 1	Year 2+ onwards
Cladribine (oral)	1.75 mg/kg per treatment course for year 1 and 2 only	107,039	107,039	-	-	3,066	980	104,105	102,019
Peginterferon beta-1a (sc)	125 mcg every two weeks	37,250	37,250	4	-	1,804	1,676	39,058	38,926
Siponimod (oral)	Initial Doses: Day 1-2- 0.25 mg once daily, Day 3- 0.5 mg once daily, Day4- 0.75 mg, and Day 5- 1.25 mg Maintenance dose: Day 6 onwards:2mg daily	88,565	88,370	-	-	1,228	754	89,793	89,124
Ofatumumab (sc)	At week 0,1,2- 20 mg sc injection, followed by subsequent monthly dosing, starting at week 4	51,325	44,502	4	-	1,204	1,204	52,534	45,706
Ozanimod (oral)	Initial 7-day titration, afterwards maintenance dosage of 0.92 mg taken orally once daily starting on day 8	48,057	48,058	360	-	1,263	908	49,680	48,966
Natalizumab (sc)	300 mg once every 4 weeks	59,914	59,914	360	-	1,204	1,204	61,478	61,118

Ref: EMA Label for all drugs; mcg: microgram; mg: milligram; kg: kilogram; sc: subcutaneous
Source: NUPCO (National Unified Procurement Company)

Table S4: Relapse costs

Cost per Relapse (SAR)	
Active RRMS	4,284
Highly Active RRMS	7,551
Aggressive RRMS	8,320

RRMS: Relapse Remitting Multiple Sclerosis

Source: KOL Insights; Samjoo IA, Worthington E, Drudge C, et al. Comparison of ofatumumab and other disease-modifying therapies for relapsing multiple sclerosis: a network meta-analysis. *J Comp Eff Res*. 2020;9(18):1255-1274. doi:10.2217/cer-2020-0122

Table S5: Adverse Event Costs and Event Rates

Adverse Event	Cost per event	Cladribine	Peginterferon beta-1a	Siponimod	Ofatumumab	Ozanimod	Natalizumab
Abdominal pain	76	-	-	-	-	-	-
Liver enzyme increased	395	-	-	5.60%	-	5.50%	-
Arthralgia/Back pain/Pain extremity	76	-	18.00%	6.00%	-	-	14.00%
Bradycardia	82	-	-	6.00%	-	-	-
Depression	453	-	-	--	-	-	-
Diarrhea	87	-	-	6.00%	-	-	-
Dizziness/Falls	0.00	-	-	18.00%	-	-	11.00%
Fatigue	0.00	-	6.50%	-	-	-	23.00%
Flu-like symptoms	76	-	32.00%	-	-	-	-
Headache	76	-	28.00%	15.00%	-	-	32.00%
Hypersensitivity including pruritis	155	11.80%	-	-	-	-	-
Hypertension	173	-	-	12.60%	-	-	-
Infections	2,938	49.00%		49.00%	51.60%	35.00%	-
Infusion-related reactions	435	-	-	-	-	-	23.10%
Injection-related reactions	0.00	-	-	-	20.60%	-	-
Leukopenia	219	-	-	-	-	-	-
Lower respiratory tract infection/Dyspnoea	922	-	-	-	-	-	-
Lymphopenia/ Decrease in IgM	219	11.30%	-	-	14.30%	-	-
Nasopharyngitis	76	-	8.00%	-	-	-	27.00%
Nausea/Vomiting	84	-	6.00%	7.00%	-	-	15.00%
Thyroid disorders	952	-	-	-	-	-	-
Upper respiratory tract infections	76	-	46.00%	-	50.30%	-	-
Urinary tract infection	495	-	5.00%	-	-	-	16.00%
**Average AE cost per drug**	**-**	**1482.878**	**130.2464**	**1515.798**	**1585.764**	**1050.177**	**247.858**

Source: KOL Insights

Table S6: Budget impact of introducing new interventions for Active RRMS Naïve with and without MEA based on 100% uptake

Scenario	With and without MEAs	Drug acquisition	Administration	Monitoring	Adverse Events	Relapse Cost	Total
Peginterferon beta-1a	-	98,344,422.32	-12,484,210.83	2,624,055.62	-16,181,997.10	-440,583.45	71,861,686.57
Siponimod	-	781,358,586.10	-12,495,336.47	12,914,829.14	23,161,189.29	-3,756,625.68	780,337,642.36
Ozanimod	No MEA	2,426,371,716.08	-11,534,081.03	4,626,671.12	-3,900,219.77	-3,164,475.29	228,665,611.12
	MEA	161,014,361.01	-11,534,081.03	4,626,671.12	-3,900,219.77	-3,164,475.29	147,042,256.05
Natalizumab	No MEA	400,925,215.08	-11,534,081.03	-2,331,750.20	-14,611,792.42	-10,862,430.47	361,585,160.95
	MEA	328,236,116.24	-11,534,081.03	-2,331,750.20	-14,611,792.42	-10,862,430.47	288,896,062.11
Ofatumumab	No MEA	213,382,781.54	-12,484,210.83	2,624,055.62	3,250,283.94	-9,382,054.47	197,390,855.80
	MEA	70,158,246.48	-12,484,210.83	2,624,055.62	3,250,283.94	-9,382,054.47	54,166,320.74
Ocrelizumab	No MEA	520,228,742.72	5,358,973.21	-5,407,767.61	8,291,925.26	-10,270,280.07	518,201,593.51
	MEA 1	344,800,358.62	5,358,973.21	-5,407,767.61	8,291,925.26	-10,270,280.07	342,773,209.41
	MEA 2	338,167,089.71	5,358,973.21	-5,407,767.61	8,291,925.26	-10,270,280.07	336,139,940.50
	MEA 3	323,939,982.96	5,358,973.21	-5,407,767.61	8,291,925.26	-10,270,280.07	32,1912,833.75

MEA: Managed Entry Agreement; Color red, orange, yellow, and green depict low to high values

*The values in the table are the difference in total cost from the Standard of Care (SOC) cost (Values of SOC: Drug acquisition: 398,968,393.88; Administration: 12,495,336.47; Monitoring: 18,293,930.82; Adverse Events: 17,920,887.05; Relapse Cost: 15,599,633.66; Total: 463,278,181.87)

Table S7: Budget impact of introducing new interventions for Highly Active RRMS Naïve scenarios with and without MEA based on 100% uptake

Scenario	With and without MEAs	Drug acquisition	Administration	Monitoring	Adverse Events	Relapse Cost	Total
Cladribine	No MEA	32,082,038.41	-5,269,451.17	-1,090,855.05	-123,707.92	-2,911,347.39	22,686,676.87
	MEA	-126,946,688.85	-5,269,451.17	-1,090,855.05	-123,707.92	-2,911,347.39	-22,090,030.38
Natalizumab	No MEA	62,190,919.90	-5,066,983.57	242,397.29	-3,596,646.59	-4,272,823.58	49,496,863.44
	MEA	46,849,729.10	-5,066,983.57	242,397.29	-3,596,646.59	-4,272,823.58	34,155,672.65
Ofatumumab	No MEA	22,689,175.79	-5,267,107.80	1,286,230.25	165,612.21	-3,705,541.84	15,168,368.61
	MEA	-8,693,172.09	-5,267,107.80	1,286,230.25	165,612.21	-3,705,541.84	-16,213,979.27
Ocrelizumab	No MEA	87,319,619.21	-1,508,828.26	-405,499.03	1,227,524.55	-4,045,910.88	82,586,905.58
	MEA 1	51,043,171.30	-1,508,828.26	-405,499.03	1,227,524.55	-4,045,910.88	46,310,457.68
	MEA 2	49,753,369.01	-1,508,828.26	-405,499.03	1,227,524.55	-4,045,910.88	45,020,655.39
	MEA 3	46,986,987.15	-1,508,828.26	-405,499.03	1,227,524.55	-4,045,910.88	42,254,273.52

MEA: Managed Entry Agreement, Color red, orange, yellow, and green depict low to high values

*The values in the table are the difference in the total cost from the Standard of Care (SOC) cost. (Values of SOC: Drug acquisition: 106,289,311.81; Administration: 5,269,451.17; Monitoring: 3,119,689.69; Adverse Events: 4,293,635.56; Relapse Cost: 6,088,125.18; Total: 125,060,213.42)

Table S8: Budget impact of introducing new interventions for Highly Active RRMS Non Naïve with and without MEA based on 100% uptake

Scenario	With and without MEA	Drug acquisition	Administration	Monitoring	Adverse Events	Relapse Cost	Total
Cladribine	No MEA	5,426,066.91	-1,679,935.63	-430,692.63	-39,438.90	-928,156.66	2,347,843.09
	MEA	-7,295,156.34	-1,679,935.63	-430,692.63	-39,438.90	-928,156.66	-10,373,380.16
Natalizumab	No MEA	19,826,873.52	-1,615,387.63	77,277.85	-1,146,634.54	-1,362,204.21	15,779,924.99
	MEA	14,976,627.25	-1,615,387.63	77,277.85	-1,146,634.54	-1,362,204.21	10,929,678.72
Ofatumumab	No MEA	7,233,458.19	-1,679,188.54	410,058.65	52,798.26	-1,181,351.06	4,835,775.49
	MEA	-3,372,406.80	-1,679,188.54	410,058.65	52,798.26	-1,181,351.06	-5,770,089.50
Ocrelizumab	No MEA	27,838,067.82	-481,024.35	-129,275.75	391,342.89	-1,289,862.95	26,329,247.65
	MEA 1	15,384,381.20	-481,024.35	-129,275.75	391,342.89	-1,289,862.95	13,875,561.03
	MEA 2	14,831,608.79	-481,024.35	-129,275.75	391,342.89	-1,289,862.95	13,322,788.62
	MEA 3	13,646,016.56	-481,024.35	-129,275.75	391,342.89	-1,289,862.95	12,137,196.39

MEA: Managed Entry Agreement; Color red, orange, yellow, and green depict low to high values

*The values in the table are the difference in the total cost from the Standard of Care (SOC) cost (Values of SOC: Drug acquisition: 33,885,730.35; Administration: 1,679,935.63; Monitoring: 994,577.55; Adverse Events: 1,368,839.20; Relapse Cost: 1,940,934.27; Total: 39,870,017.01)

Table S9: Budget impact of introducing new interventions for Aggressive RRMS Naïve scenarios with and without MEA based on 100% uptake

Scenario	With and without MEAs	Drug acquisition	Administration	Monitoring	Adverse Events	Relapse Cost	Total
Natalizumab	No MEA	27,583,743.72	-2,419,489.27	274,094.73	-1,718,286.45	-2,296,759.73	21,423,302.99
	MEA	20,477,225.86	-2,419,489.27	274,094.73	-1,718,286.45	-2,296,759.73	14,316,785.14
Ofatumumab	No MEA	9,264,363.01	-2,512,299.14	758,184.07	26,503.62	-2,005,799.15	5,530,952.42
	MEA	-5,921,487.64	-2,512,299.14	758,184.07	26,503.62	-2,005,799.15	-9,654,898.23
Ocrelizumab	No MEA	39,237,4,62.30	-769,354.49	-26,374.52	518,977.49	-2,180,375.50	36,780,335.28
	MEA 1	22,236,145.82	-769,354.49	-26,374.52	518,977.49	-2,180,375.50	19,779,018.80
	MEA 2	21,609,670.42	-769,354.49	-26,374.52	518,977.49	-2,180,375.50	19,152,543.40
	MEA 3	20,265,999.23	-769,354.49	-26,374.52	518,977.49	-2,180,375.50	17,808,872.21

MEA: Managed Entry Agreement; Color red, orange, yellow, and green depict low to high values

*The values in the table are the difference in the total cost from the Standard of Care (SOC) cost (Values of SOC: Drug acquisition: 50,550,869.74; Administration: 2,513,385.91; Monitoring: 1,285,111.15; Adverse Events: 2,041,522.98; Relapse Cost: 3,227,833.60; Total: 59,618,723.37)

Table S10: Budget impact of introducing new interventions for Aggressive RRMS Non Naïve with and without MEA based on 100% uptake

Scenario	With and without MEAs	Drug acquisition	Administration	Monitoring	Adverse Events	Relapse Cost	Total
Natalizumab	No MEA	3,792,415.77	-332,649.16	37,684.56	-236,242.65	-315,775.40	2,945,433.11
	MEA	2,822,342.99	-332,649.16	37,684.56	-236,242.65	-315,775.40	1,975,360.34
Ofatumumab	No MEA	1,273,732.70	-345,409.35	104,240.72	3,643.91	-275,772.00	760,435.98
	MEA	-1,871,066.24	-345,409.35	104,240.72	3,643.91	-275,772.00	-2,384,362.96
Ocrelizumab	No MEA	5,394,654.63	-105,776.51	-3,626.16	71,352.84	-299,774.04	5,056,830.75
	MEA 1	2,903,917.30	-105,776.51	-3,626.16	71,352.84	-299,774.04	2,566,093.42
	MEA 2	2,793,362.82	-105,776.51	-3,626.16	71,352.84	-299,774.04	2,455,538.94
	MEA 3	2,556,244.37	-105,776.51	-3,626.16	71,352.84	-299,774.04	2,218,420.50

MEA: Managed Entry Agreement; Color red, orange, yellow, and green depict low to high values

*The values in the table are the difference in the total cost from the Standard of Care (SOC) cost (Values of SOC: Drug acquisition: 6,950,105.01; Administration: 345,558.76; Monitoring: 176,686.52; Adverse Events: 280,683.58; Relapse Cost: 443,786.28; Total: 8,196,820.16)
